# Control of Obesity, Blood Glucose, and Blood Lipid with *Olax imbricata* Roxb. Root Extract in High-Fat Diet-Induced Obese Mice

**DOI:** 10.1155/2022/7781723

**Published:** 2022-09-02

**Authors:** Thi Nga Vo, Thi-Diem-My Luong, Thi-Phuong-Hoa Le, Khanh Son Trinh

**Affiliations:** Faculty of Chemical and Food Technology, Ho Chi Minh City University of Technology and Education, Ho Chi Minh City 720300, Vietnam

## Abstract

Mice were used in *in vivo* experiments to evaluate the effects of doses of *n*-hexane extract (from 100 to 1,300 mg/kg body weight/day) on the ability to control obesity, blood glucose, and blood lipid. In this study, body weight gain, caloric intake, glucose tolerance, blood lipid, histopathological study, and locomotion activity were examined. Furthermore, this study evaluated the lethality of the extract in extremely high doses in the tested mice. After 3 months of use with an extremely high dose of 5,000 mg/kg body weight/day (equivalent to 350 g/day for a 70 kg person), no animals with abnormal conditions or death were observed. This initially demonstrated the safety of the extract. In addition, after 6 weeks of testing on high-fat diet-induced obese mice, *n*-hexane extract at a dose of 500 mg/kg body weight/day (equivalent to 35 g/day for a 70 kg person) demonstrated a positive effect on the ability to control obesity, blood glucose, and blood lipid through the results of body weight, blood lipids, glucose tolerance, and histopathology (white fat, liver, and kidney tissues). In this study, *n*-hexane extract from the roots of Duong-dau tree has proven to be strongly biologically active in preventing and supporting the treatment of diseases related to overweight and obesity, helping to control blood glucose levels thereby reducing the risk of type 2 diabetes.

## 1. Introduction

Obesity and overweight are defined as excessive accumulation of body fat that is harmful to health. Obesity and overweight are not only common in high-income countries but are also now showing signs of increasing in low- and middle-income countries, especially in large cities. In most parts of the world, obesity and overweight are more deadly than underweight, with the exception of parts of sub-Saharan Africa and Asia. The main cause of obesity and overweight is the imbalance between calories consumed and calories expended. This is due to (i) the use of energy-rich foods high in fat and sugar (or starch) and (ii) less physical activity [1].

Obesity can be reduced in three ways: expanded energy (energy expended is higher than the energy absorbed from food), appetite suppression, and inhibition of digestive enzymes. In the diet, carbohydrates (starch) are a large amount and the main source of energy for the body. In the body, starch is digested into monosaccharides by *α*-amylase and *α*-glucosidase. Therefore, inhibiting these enzymes can help reduce starch digestion and the amount of energy absorbed into the body [2]. Previous studies have also shown that *α*-glucosidase inhibitors (AGIs) help inhibits/restricts carbohydrate absorption from the gut and can be used to treat patients with type 2 diabetes (DM2) or impaired glucose tolerance (IGT). For patients with IGT, AGIs may prevent or delay the onset of DM2 [3].

Obesity and being overweight increase the risk of several diseases. It induces several harmful metabolic effects on blood pressure, blood lipids, and insulin resistance and increases the risk of ischemic stroke, heart disease, and DM2. They also increase the risk of breast, colon, prostate, endometrium, kidney, and gallbladder cancers. Previous studies have shown that these are completely preventable and controllable risks. Most AGIs are derived from synthetic chemicals that need to be approved by government agencies and often have side effects. Therefore, there is a need for alternative therapies with little or no side effects to control overweight, obesity, and other related diseases. Many traditional herbal medicines can control obesity and overweight, especially those with active AGIs [4].

*Olax* is the largest genus of Olacaceae with about 40 species, and has been of interest to researchers for its biological activities. Nwaigwe et al. [5] studied methanol extract from the roots of *Olax viridis* with the ability to protect the liver, and against acetaminophen causing liver damage. Sule et al. [6] also reported on the ability to treat fever and jaundice with acetone extract from *Olax mannii*.

*Olax imbricata*, in Vietnamese as Duong-dau, has been used as a traditional herbal medicine for DM2 and cancer in Phu Yen province, Vietnam, where this plant is widely distributed [7]. However, the chemical composition and biological activity of Duong-dau plant have not been elucidated by many studies. et al. [8], in Thailand, detected polyphenols, flavonoids, saponins, tannins, and alkaloids in *Olax imbricata*. Theoretically, these are compounds known for their antibacterial and antioxidant effects.

Detailed studies on the chemical composition of Duong-dau root were performed on ethyl acetate and methanol extracts. As a result, three acetylenic fatty compounds were isolated from ethyl acetate extract [9]. From the methanol extract, 13 compounds were isolated, including three phenolic compounds [10], three phenolic glycosides [11], three derivatives 1,2,3,4-tetrahydronaphthalene and a tropolone derivative [7], and three triterpenoid glycosides [12]. Nine purified compounds isolated from the Duong-dau roots were tested for their AGI activity. The results showed that olaximbriside D, a naphthalene derivative, moderately exhibited an AGI activity with an IC_50_ value of 217.50 *µ*g/mL (545.86 *µ*M); two acetylenic fatty acids and a triterpenoid glycoside, 28-O-*β*-D-glucopyranosyl oleanolate, exhibited fairly strong activity with IC_50_ values of 3.15 *µ*g/mL (10.10 *µ*M), 29.64 *µ*g/mL (92.63 *µ*M) and 34.75 *µ*g/mL (56.15 *µ*M), respectively. The three later compounds were stronger inhibitors of *α*-glucosidase compared to acarbose, a common commercial drug used to treat diabetes.

According to Nakao et al. [13], polyacetylenic acids, often derived from marine algae, exhibit a wide range of biological activities ranging from cytotoxic, antibacterial, and antifungal, to enzyme inhibition. Polyacetylenic acid compounds from the marine algae *Callyspongia truncate* showed strong AGI activity, e.g., callyspongynic acid, corticatic acid A, and Petrosynol were able to inhibit *α*-glucosidase with IC_50_ of 0.25, 0.16, and 4.08 *µ*g/mL, respectively. Compared to other compounds, it is possible that the carboxylic acid and allylic alcohol functional groups connected to acetylene play an important role in the activity [13]. This explains the AGI ability of two acetylenic fatty acids found in Duong-dau root.

To evaluate the *α*-glucosidase inhibitory activity of different triterpenoid derivatives, López et al. [14] performed on *α*-amyrin, *β*-amyrin, ursolic acid, oleanolic acid, and betulinic acid compounds. The IC_50_ values of these compounds were 1.45, 0.02, 1.08, 0.98, and 2.37 *µ*M, respectively. This suggests that the oleanane-type triterpenoid, represented by *β*-amyrin and oleanolic acid, exhibits higher AGI activity than other frameworks. According to another approach, the study to evaluate the structural relationship and AGI activity of triterpenoid glycosides was performed by Dou et al. [15]. Accordingly, when oleanolic acid carries the glucuronic acid unit at C-3 or the glucose moiety at C-28 of the aglycone, these functional groups play an important role in *α*-glucosidase inhibition. Besides, the addition or replacement of the glucose moiety by other sugar units may cause a loss of this inhibitory activity. This explains the AGI activity of 28-O-*β*-D-glucopyranosyl oleanolate over other triterpenoid compounds isolated from Duong-dau root. In addition, the AGI activity of 28-O-*β*-D-glucopyranosyl oleanolate was also explained through molecular docking [16].

From the chemical composition, related to *in vitro α*-glucosidase inhibitory activity, these results have initially contributed to demonstrating the ability to control blood sugar and diabetes from the folk experience of local people of Phu Yen province, Vietnam. The acetylenic fatty acid compounds have been isolated from the ethyl acetate extract of Duong-dau root. These compounds are lipophilic and well soluble in less polar solvents such as *n*-hexane. Although the chemical composition of *n*-hexane extract has not been elucidated, due to the less polarity of the solvent, this extract may contain fewer polar components such as fatty acids, glycerolipid, steroids, triterpenoids, etc., in which acetylenic fatty acids are specific metabolites of this plant.

Previously, there were many studies on bioactive compounds for the treatment of type 2 diabetes, overweight, and obesity. *Olax imbricata* also contains many AGIs. However, studies on AGI activity mainly have been performed *in vitro* and no study has yet reported the in vivo prevention/anti-overweight, obesity, and type 2 diabetes activity of *Olax imbricata*. Therefore, in this study, we evaluated the ability to prevent obesity and overweight, and control blood glucose and blood lipids using *n*-hexane extract from Duong-dau (*Olax imbricata*) root in high-fat diet-obese induced mice.

## 2. Materials and Methods

### 2.1. Duong-Dau Root Extract Preparation

**Figure 1:**
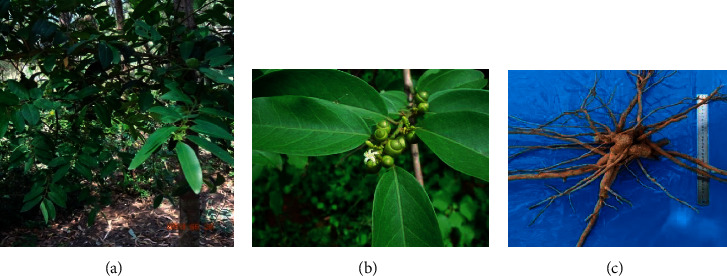
(a) Duong-dau tree, *Olax imbricata*; (b) Duong-dau flower and fruit; (c) Duong-dau root.

Duong-dau root was collected in Dong Hoa district, Phu Yen province, Vietnam, in August 2020. It was authenticated as *Olax imbricata* Roxb. (Olacaceae) by Mr. Hoang Xuan Lam from Middle Vietnam Research and Manufacturing Organic Medicinal Herb Centre in Phu Yen province, Vietnam. A voucher specimen coded as UTE–A002 was deposited at the Faculty of Chemical and Food Technology, Ho Chi Minh City University of Technology and Education, Vietnam. Duong-dau tree and its flower, fruit, and root are shown in Figure 1.

Duong-dau root extract was prepared according to a published extraction method [17]. After harvesting, Duong-dau roots were cleaned with water to remove impurities. Then, the roots were chopped and dried by a convection drier (40°C) to reach the final moisture of 8%. Next, the roots were finely ground and sieved (40 mesh). Duong-dau root powder was hot-extracted with methanol at the boiling point of the solvent in a reflux extraction system with a powder: solvent ratio of 1 : 10 (w/v). The mixture was boiled for 3 h, allowed to cool, filtered to collect the extract, and then replaced with fresh solvent. This extraction procedure was repeated in triplicates. All extracts (from three extractions) were collected. The solvent was removed by a vacuum evaporator (40°C) to obtain the methanolic extract. The extract was spread and dried by a convection dryer (50°C) to completely remove residual methanol. The extract was then thoroughly mixed with *n*-hexane, settled, and collected as the supernatant. This *n*-hexane mixing step was repeated several times until the supernatant was completely clear. The *n*-hexane supernatant was collected and *n*-hexane was removed by a vacuum evaporator (40°C). The extract was spread and dried by a convection dryer (50°C) to completely remove the *n*-hexane residue. The final extract was stored in a sealed amber-glass bottle (10°C) for further studies.

### 2.2. *In Vitro α*-Glucosidase Inhibition

*In vitro α*-glucosidase inhibition was performed according to the method of Apostolidis et al. [18] with slight modifications. The sample (in triplicate) was dissolved in dimethyl sulfoxide at different concentrations for the *α*-glucosidase inhibitory assay. A total reaction volume of 200 *μ*L/well°×°96 wells was done. The components in each well included 0.1 M phosphate buffer solution (60 (*μ*L, pH 6.8), *α*-glucosidase (20 (*μ*L, 0.3 IU/mL), sample solution (20 *μ*L), and 200 mM p-nitrophenyl *α*-D-glucopyranoside (100 (*μ*L). The reaction was carried out at 37°C for 30 min. The reaction was stopped by the addition of 50 mM NaOH solution (50 *μ*L). Negative control was done by replacing the sample with a buffer solution. Positive control was done using acarbose as a sample. A Bio Tek microplate reader is used to measure the absorbance of the samples.

(1) $$\begin{array}{c} I\left(\%\right)=\frac{A_{control}-A_{sample}}{A_{control}}\times 100, \end{array}$$

where A_control_ and A_sample_ were the absorbances (405 nm) of the control and sample, respectively. The IC_50_ (the half-maximal inhibitory concentration) was determined using the calibration curve equation between I (%) and sample concentration.

### 2.3. Animals and Husbandry

Experimental animals were 6-week-old male mice (*Mus musculus* var *albino*) with a weight of 28 ± 1 g provided by the Pasteur Institute in Ho Chi Minh City, Vietnam. Mice were acclimatized before testing with a CT diet (Table 1) at 30°C, 50–60% RH with 12/12 h light-dark cycle [20] until reaching a body weight of about 30 ± 1 g/individual (about one week). Unlimited food and drinks were provided. Experimental procedures obtained ethical clearance with reference No. IRB-A-2020 (Institutional Review Board at Dinh Tien Hoang Institute of Medicine has the operating code as IRB-VN02010 issued by the Vietnam Ministry of Health on 15^th^ October 2015).

After acclimatization, mice were fed a high-fat diet (HFD) (Table 1) for 2 weeks. Then, mice were randomly divided into 7 groups (5 individuals/group) and fed according to Table 1 (from week 3 to week 6): CT, HFD, HF-A100, HF–H100, HF–H500, HF –H900, and HF–H1300. Mice were fed twice per day. Before diet feeding, the extract (in a drop of olive oil, 50 (mL) and acarbose (in water, 50 mL) were fed to mice. The sample size was calculated following a previous study [21]. The energy of diets was calculated based on the Atwater conversion factors: protein (4 kcal/g), fat 37 (9 kcal/g), and carbohydrate (4 kcal/g) [22].

### 2.4. Bodyweight Gain (BWG) and Caloric Intake (CI)

Changes in body weight of tested groups were measured every week. Before measurement, the mouse was fasted overnight (without water feeding) [23]. The apparatus used for determining body and organ weights was a Precision Balance Precisa LS 2200C (Scales and Measuring Instruments, Germany). The difference in the mouse's body weight was calculated as its body weight gain (BWG). Furthermore, the food efficiency ratio (FER) was calculated as follows:

(2) $$\begin{array}{c} FER\left(\frac{g}{kcal}\right)=\frac{BWG}{CI}\times 100. \end{array}$$

### 2.5. Glucose Tolerance Test

The glucose tolerance test determined the postprandial glucose level in the plasma after a meal [24]. Mice were fasted for 16 h and then fed glucose (0.5 mL, 7.5%) or autoclaved rice flour (0.5 mL, 7.5%, w/v) via an oral ZONDE needle. After eating, a drop of blood was taken from the tail vein of the mouse at 0, 30, 60, 90, 120, 150, 180, and 240 min [25, 26]. Blood (a drop of whole blood) glucose level was measured with an Accu-Chek Active Glucose System (Roche Ltd., Basel, Switzerland). Furthermore, the Origin 8.5.1 software (OriginLab Corporation, Northampton, USA) was used to calculate the incremental area under the glycemic response curve (AUC).

### 2.6. Blood Biochemistry and Histopathological Examination

A volume (around 1.0 mL) of blood was collected using the cardiac puncture technique [26–28]. Using the serum of blood, levels of total cholesterol (TC), triglycerides (TG), high-density lipoprotein cholesterol (HDL), and low-density lipoprotein cholesterol (LDL) were measured by the AU480 Chemistry Analyzer (Beckman Coulter, Inc., United States).

Liver, kidney, and white adipose tissues were collected, fixed in 10% formalin solution, and embedded in paraffin. A four *µ*m-thick serial section was prepared and stained with hematoxylin and eosin (H&E) for light microscopic examination [29].

### 2.7. Locomotion Activity

In the open fields, two 12 × 12 × 12 (inches) acrylic chambers, one mouse per chamber was analyzed. The MouseActivity code and MathLab R2019 software were used to analyze the mouse locomotion activity [30].

### 2.8. Statistical Analysis

All experiments were performed in triplicate and using SPSS software, the one-way analysis of variance (ANOVA) was used to examine the data. Ducan's multiple range test (*p* < 0.05) was used to assess the differences between mean values. Values were expressed as means ± SD.

## 3. Results and Discussion

### 3.1. Solvent Residues

The solvent residues of methanol and *n*-hexane (based on AOAC 968.09 [31]) in the final extract were 190 mg/kg and 10 mg/L, respectively. Thus, residues of solvents are within the safe range for the body [32–34].

### 3.2. Body Weight Gain and Caloric Intake

**Figure 2:**
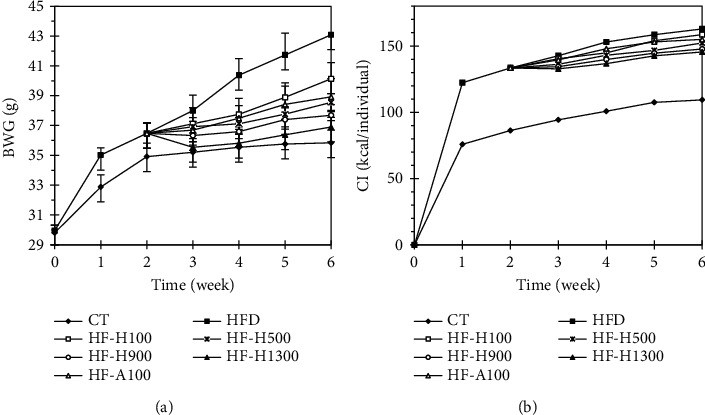
(a) Body weight gain (BWG, mean ± standard deviation, *n* = 5) and (b) caloric intake (CI, mean, *n* = 5) of tested groups.

The body weight of mice at the start of the experiment (week 0) was all the same (∼30 g/individual) (Figure 2). After one week of feeding, both the BWG and CI of the HFD group were much higher than those of the CT group. It was noted that after one week of using extract and acarbose, groups of mice had a slow increase or decrease in BWG (especially in the HF–H1300). In addition, the groups of mice might have abdominal distension, fullness, and diarrhea, depending on the amount of extract used. Fortunately, the abovementioned situation gradually disappeared the next time. This phenomenon has also been described in a previous study [35]. At the end of the first two weeks, the groups using the HFD were divided into groups: HF-A100, HF–H100, HF–H500, HF–H900, and HF–H1300. From this time until the end of week 6, the BWG groups of mice gradually differentiated and had obvious differences. Obviously, after 6 weeks of the experiment, the body weights of the groups of mice were ranked in the following order: HFD > HF-H100 > HF-A100 > HF-H500 > HF-H900 > HF-H1300 >  CT. Obviously, the BWG of the HFD group was much higher than that of the CT, HF-A100, and HF-Hs groups. At the same time, the higher the extract dose used in the HF-Hs groups was, the lower the BWG was and vice versa. Similar results were also reported by many previous studies [36, 37]. Correspondingly, at week 6, the food efficiency ratio (FER, g/kcal) of the HFD, HF-H100, HF-A100, HF-H500, HF-H900, HF-H1300, and CT groups was 26.45, 25.89, 25.11, 25.31, 25.51, 25.36, and 32.78, respectively. A high FER value means that a large amount of energy from the feed has been absorbed into the body and converted into the body weight of the test animal. Thus, in this study, high-fat diets increased body weight but decreased the food efficiency ratio of experimental animals. In addition, the HFD diet accompanied by Duong-dau root extract or acarbose reduced BWG and increased FER.

*In vitro* test results showed that Duong-dau root extract has an *α*-glucosidase inhibitory effect. Specifically, the IC_50_ values (*µ*g/mL) of the Duong-dau extract and acarbose were 47.8 ± 0.3 and 135.4 ± 0.2, respectively. Thus, Duong-dau extract has the ability to inhibit *α*-glucosidase enzyme higher than that of acarbose (2.83 times). Previous studies also showed that extracts from some plants were more effective than acarbose in terms of *α*-glucosidase inhibitory [38]. In other words, this extract acts as an *α*-glucosidase inhibitor (AGI). Normally, carbohydrates in the diet are completely digested and absorbed in the small intestine by the action of *α*-glucosidase (EC. 3.2.1.20), which breaks down starches and disaccharides into glucose. In the presence of AGIs, carbohydrates are not fully digested or digested slowly because the enzyme *α*-glucosidase (in the jejunum and ileum) is inhibited. When enzyme activity in the small intestine is reduced, the unabsorbed carbohydrate continues to move to the large intestine, where bacteria convert carbohydrates into short-chain fatty acids, hydrogen, CO_2_, and methane [39]. As a result, the truly caloric intake (from carbohydrates) and body weight of the experimental groups that used AGI (HF-Hs and HF-A100) were lower than those of the control groups that did not use it.

### 3.3. Postprandial Blood Glucose Level (PBL)

**Figure 3:**
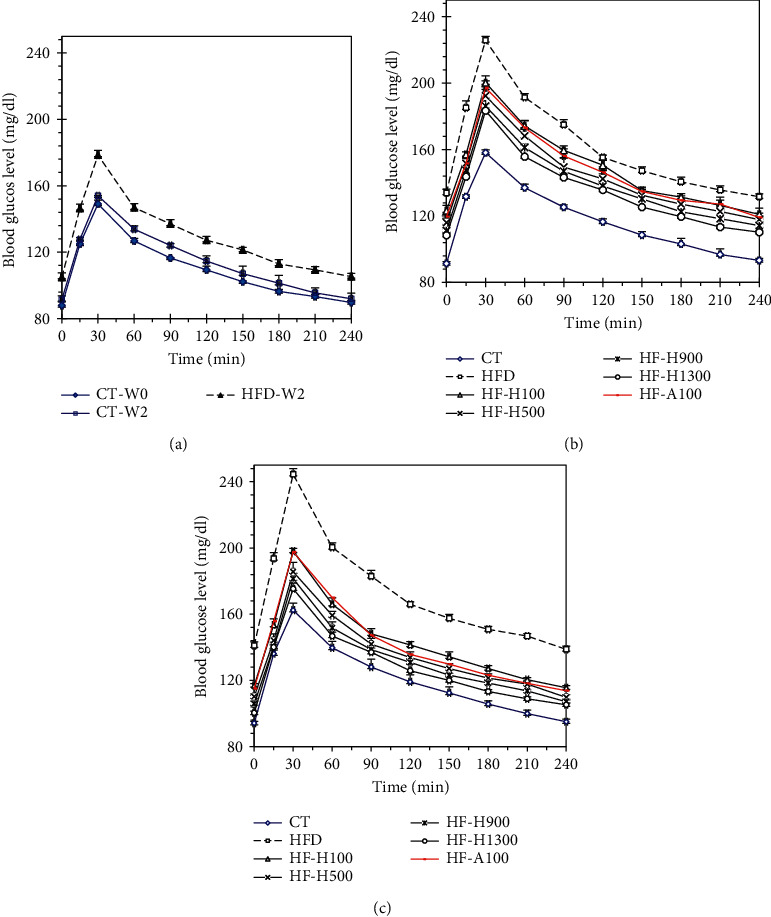
Postprandial blood glucose level (PBL) in mice at the end of (a) week 0 (W0) and week 2 (W2); (b) week 4; and (c) week 6 of experiments (data were shown in mean ± standard deviation, *n* = 5).

Figure 3 shows the PBL of tested groups at weeks 0, week 2, week 4, and week 6. In all cases, after feeding glucose (0.5 mL, 7.5%), blood glucose concentration increased rapidly and reached PBLmax at 30^th^-min. Thereafter, blood glucose levels gradually decreased in all experimental groups. After 240 min, the glucose level returned to the fasting (at 0 min) blood glucose level (FPBL) [40].

In the case of the CT group, the curves of PBL were almost similar during 6 weeks of testing. In contrast, in the case of the HFD group, FPBL and PBLmax increased gradually from week 0 to week 6. From week 2 to week 6, all PBL values of the HFD group were higher than that of CT group. The PBLmax differences between the HFD and CT groups at weeks 2, 4, and 6 were 24.54, 67.65, and 82.11 (mg/dl), respectively.

**Figure 4:**
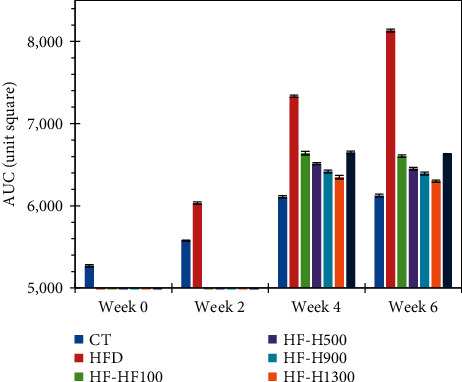
The area under curve (AUC) of PBL after feeding of glucose (data were shown in mean ± standard deviation, *n* = 5).

In the case of HF-Hs, based on the measurement results at weeks 4 and 6, all values on the curves of PBL could be arranged in the following order: HFD > HF-H100 > HF-A100 > HF-H500 > HF-H900 > HF-H1300 > CT.  Figure 4 also shows that the UACs of the experimental groups were also arranged in the abovementioned order.

All of the data mentioned above suggested that HFD had the potential to increase the risk of impaired glucose tolerance (IGT). The longer HFD was used, the lower the control of blood glucose and the higher the postprandial blood sugar was. From Figures 3 and 4, the control of blood glucose of Duong-dau extract could be clearly observed. In particular, at a dose of 100 mg/kg BW/day, the effects of the extract and acarbose were quite similar. The higher dose and the longer the using duration was, the better the ability to control blood sugar was. Interestingly, Figures 3 and 4 are obtained from mice fed glucose (instead of starch). This means that the above glycemic control can still be effective even if starch is not present in the diet. In other words, the effectiveness of Duong-dau root extract and acarbose is not merely a direct inhibitory effect on *α*-glucosidase.

In this study, both *in vitro* and *in vivo* experimental results showed that the extract had a good ability to inhibit the activity of *α*-glucosidase, which slowed down carbohydrate breakdown and lowered blood glucose levels. Based on an *in vivo* experiment, William-Olsson [41] showed that acarbose (a type of AGI) has the ability to prevent obesity and slow lipid accumulation.

Some other studies also showed that mixed extract containing *Platycodon grandiflorum*, *Apium graveolens*, and green tea or green coffee bean extracts has anti-obesity and decreasing body fat effects [4, 37]. et al. [2] stated antioxidants compounds from many plant extracts act as AGIs. Moreover, AGIs can help reduce starch digestion, reducing the amount of energy absorbed into the body. This result is similar to that of Shen et al. [42] using *Grifola frondosa* nonpolar bioactive components on HFD and streptozotocin-induced hyperglycemic mice. According to the International Diabetes Federation [43], AGIs in combination with insulin, metformin, and sulfonylureas are the best treatment for IGT in patients with MD2. van de Laar [3] stated AGIs inhibit/restrict carbohydrate absorption from the gut and can be used to treat patients with DM2 or IGT. For patients with IGT, AGIs may prevent or delay the onset of type 2 diabetes. Not only inhibiting *α*-glucosidase activity, a number of other studies have demonstrated that AGIs also stimulate the secretion of glucagon-like peptide-1 (GLP1), which helps lower postprandial blood sugar by stimulating insulin secretion and inhibiting glucagon secretion [44, 45]. GLP-1 is secreted by L cells of the ileum when it senses nutrients entering the ileum. GLP-1 acts as one of the hormones that inhibit the peristalsis to increase the time used for nutrients to be absorbed into the body [46]. Inhibitors slow down the digestion of polysaccharides leading to an increase in the number of polysaccharides entering the ileum. Thus, these polysaccharides are not absorbed by the body and at the same time, the peristalsis is inhibited, so they are stored in the ileum for a longer time than usual and stimulate the secretion of GLP-1 [45]. When GLP-1 is secreted more, insulin is also stimulated to secrete more and thereby reduce blood glucose content [44]. The abovementioned mechanisms help explain why the above glycemic control results are effective even in the absence of starch in the diet.

### 3.4. Blood Lipid Parameters

According to Table 2, after 6 weeks of testing (i.e., after 4 weeks of using extract or acarbose), the TG, TC, LDL, TG/HDL, TC/HDL, and LDL/HDL values of the HFD were the highest and these of the HF-H1300 were lowest compared to the others. Obviously, the higher the dose used, the lower these values were. In addition, the change in HDL values tended to be opposite to that of LDL values of each group. The aforementioned trends have also been described in some previous studies [47–50]. The results from Table 2 also showed that Duong-dau root extract is more effective than acarbose in controlling blood lipids.

Insulin deficiency leads to a strong mobilization of stored energy, leading to lipolysis in adipocytes and the release of free fatty acids. This is because glucose, provided by food, is not sufficient for the body's energy production [51]. If free fatty acids are present in large quantities in the blood, they are transported to the liver, leading to an increase in VLDL, which inhibits lipoprotein lipase in peripheral tissues. Lipoprotein lipase is an enzyme that hydrolyzes TG in lipoproteins. If this enzyme is inhibited, it reduces the breakdown of TG in the blood, causing TG levels to increase [52]. The results of our study show that using Duong-dau extract not only reduced postprandial hyperglycemia but also reduced TG levels in the blood, which was caused by increased stimulation of insulin secretion.

The decrease in TG/HDL index showed that Duong-dau extract reduced insulin resistance and increased insulin secretion [53, 54]. Lipoprotein enzyme activity is reduced in the presence of insulin resistance despite the absence of type 2 diabetes. Insulin stimulates the secretion of the enzyme lipoprotein lipase. Lipoprotein lipase helps to hydrolyze triglycerides contained within lipoproteins. During TG degradation of chylomicrons and VLDLs, residual chylomicrons are converted to HDLs. Insulin resistance not only increases TG but also decreases HDL [55]. Insulin can directly stimulate the catabolism of LDL in the body. This insulin-induced enhancement of LDL catabolism will lower blood LDL levels. Lowering LDL reduces the risk of vascular disease in patients with diabetes [56]. Thus, according to the results of this study, Duong-dau root extract has the effect of stimulating insulin secretion, thereby increasing the catabolism of LDL and reducing the concentration of LDL in the blood [53, 54]. At the same time, this extract helps to increase HDL levels through increased secretion of lipoprotein lipase. Increasing HDL levels reduces the risk of developing atherosclerosis in patients with diabetes [57].

A high TC/HDL ratio increases the risk of cardiovascular disease in patients with diabetes [58]. Lipid metabolism disorders lead to glucose metabolism disorders. High blood sugar is a direct cause of elevated TG and high TC in the blood. Hyperlipidemia is also a major factor accelerating atherosclerosis and vascular complications in diabetes [36]. Thus, using Duong-dau extract has the effect of reducing TC/HDL thereby reducing cardiovascular complications caused by diabetes.

### 3.5. Weight of Tissues

The results from Table 3 showed the HFD group has the highest ratio (%) of LW/BW, KW/BW, and AW/BW, compared with those of the other groups. The ratios of LW/BW and KW/BW of the remaining groups were not statistically different (*p* < 0.05). Thus, the HFD group had body weight, and ratios of liver tissue, kidney tissue, and adipose tissue to body weight were significantly higher than those of the other groups. Specifically, the liver weight of the HFD group increased significantly (23.4%) compared with that of the CT group. The LW/BW ratio of the HFD group was significantly higher (6.7%, *p* < 0.05) compared with that of the CT group. In addition, the results showed the AW/BW ratio was inversely proportional to the dose of the used extract. This ratio was the same for the HF-A100 and HF-H100 groups. This means that the extract had the ability to reduce body fat. The higher the dose of extract was, the higher this effect was. Actually, many previous studies have demonstrated that increasing the dosage of herbal extracts will positively impact enhancing health conditions [36, 40].

### 3.6. Histopathological Study of White Adipose Tissues

**Figure 5:**
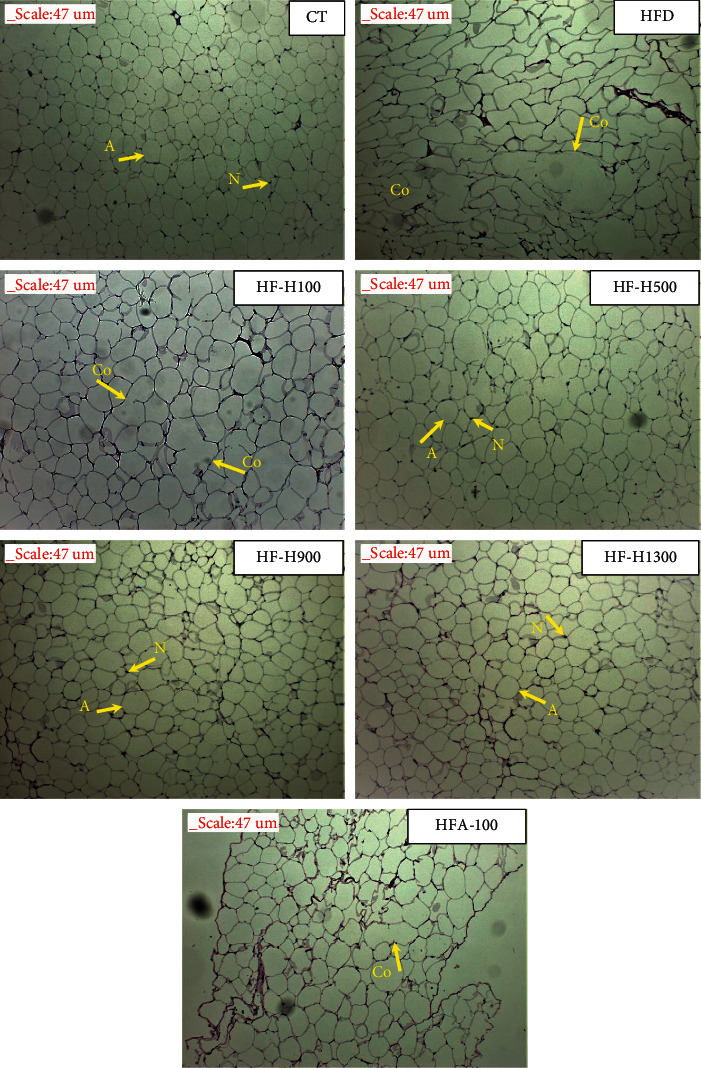
Photomicrographs of white adipose tissue (A: adipocytes; Co: coalescence; and N: nucleus).

According to Figure 5, adipocyte size was different between the tested groups. Adipocytes in the HFD group were coalescent and larger than those in the CT group [37]. Adipocytes of the HFD group also had the phenomenon of pinching each other, causing cell deformation. The HF-Hs and HF-A100 groups had smaller adipocytes and less coalescence than those of the HFD group. Moreover, the HF-Hs groups tended to gradually decrease the size of adipocytes and coalescence with increasing doses of Duong-dau extract. The size of adipocytes of HF-A100 and HF-H100 were nearly similar, but the HF-H100 group still had coalescence. From the abovementioned results, it is shown that both Duong-dau extract and acarbose have the effect of reducing both size and coalescence of adipocytes [36, 37].

The lipid droplet is composed of a monomeric phospholipid layer, a neutral lipid core, and binding proteins. The main components of the neutral lipid core are triglycerides (TG) and cholesterol esters (SE). In the HFD diet, the TG content in the lipid droplet was high, causing its size to increase. When the amount of TG in the lipid droplet is high, it is overloaded in storage, eventually breaking the cell wall. Using some plant extracts can reduce the amount of energy absorbed into the body, thus preventing the loss of cell walls in adipose tissue [59]. When the body lacks energy, it will use lipids stored in tissues, breaking down TG into free fatty acids (FFAs). Next, FFAs are transported intracellularly to mitochondria and oxidized to form ATP molecules. When TG is degraded, the size of lipid droplets is reduced [59]. Thus, Duong-dau root extract inhibits the activity of *α*-glucosidase, reducing the amount of glucose absorbed into the body. To replenish the energy deficit from glucose, the body enhances the breakdown of TG and ultimately reduces the weight of adipose tissue and the size of lipid droplets in the adipose tissue.

### 3.7. Histopathological Studies of Liver Tissues

**Figure 6:**
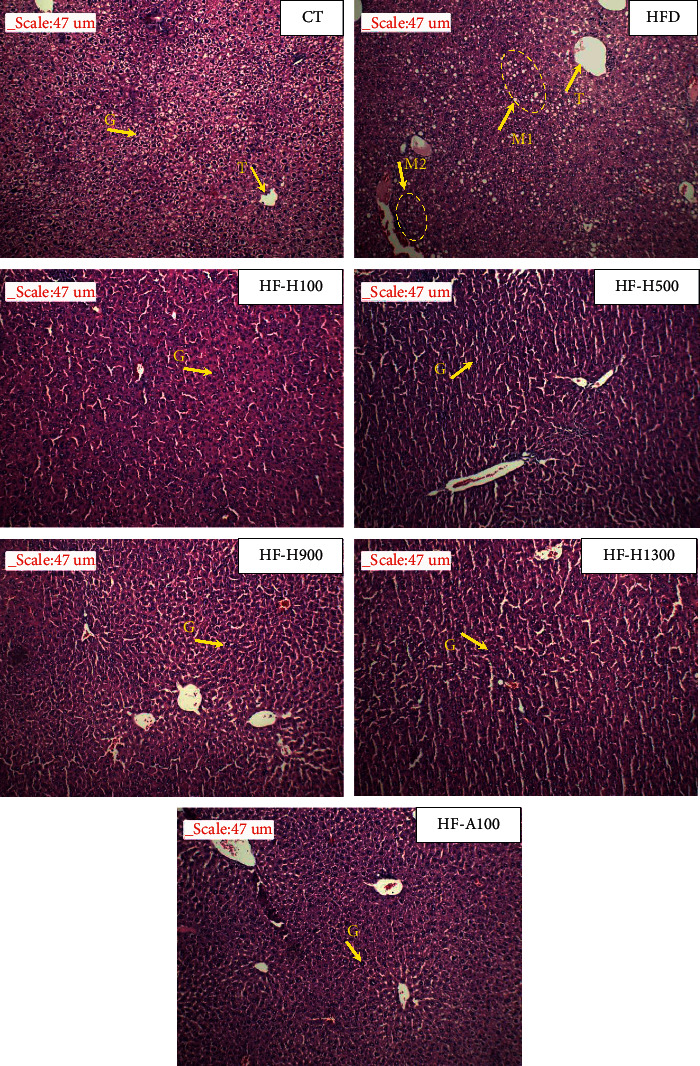
Photomicrographs of liver tissues (T: central vein; G: hepatocytes; M1: macrosteatosis; and M2: microsteatosis).

As shown in Figure 6, liver tissue of the HFD group showed a dense appearance of macrosteatosis (M1) and microsteatosis (M2). Meanwhile, there was no appearance of lipid droplets in the liver tissue samples of the HF-Hs, HF-A100 groups even though the mice of these groups were fed high-fat diets. The liver tissue samples of these groups were similar to those of the CT group. Some studies showed that the lipid droplets in the liver of the HFD group are larger than that of the CT group [37]. Thus, it can be concluded that the extract was effective in preventing the formation of lipid droplets in the liver induced by the high-fat diet.

Consumption of a high-fat diet significantly increased the levels of triglycerides (TG), total cholesterol (TC), and nonesterified fatty acids (NEFAs) in the liver [60]. Thus, Duong-dau root extract could reduce lipid accumulation in the liver that helps to reduce liver mass, number, and size of lipid droplets. In addition, some studies have shown that hyperglycemia (in the case of diabetes) leads to an overproduction of free radicals, and these free radicals have a deleterious effect on cellular function, causing them to vulnerable to oxygen stress [57]. The results of this study suggest that this extract may help control hyperglycemia, reduce oxidative stress, and may ultimately prevent the development of nonalcoholic fatty liver disease (NAFLD) [60].

### 3.8. Histopathological Studies of Kidney Tissues

**Figure 7:**
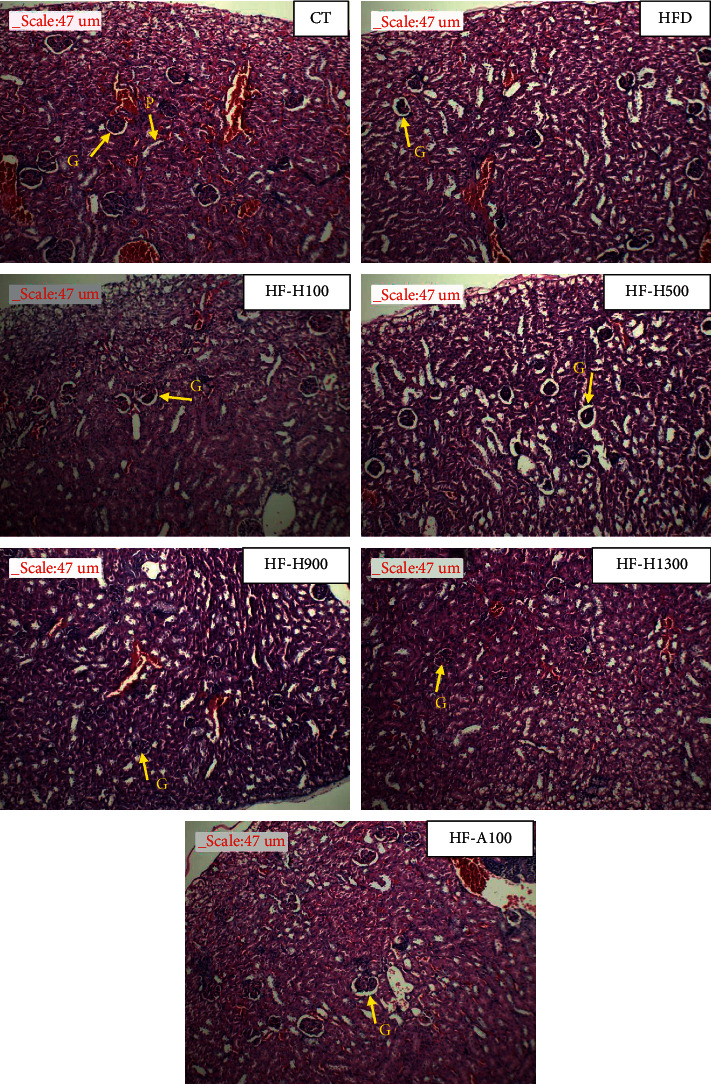
Photomicrographs of key tissues (G: glomerulus; P: proximal convoluted tubule).

Figure 7 shows that there was a difference between the experimental groups. Histological studies tested groups showed increased glomerular size and significant hydropic changes in the proximal convoluted tubules. These changes could be ordered as HFD > *A*100≈HF-H100≈HF-H500 > HF-H900 > HF-H1300. These alterations were effectively decreased post-treatment with the increased doses of the extract. These results again suggest the protective action of Duong-dau root extract in diabetic renal injury [61, 62].

### 3.9. Locomotion Activity

**Figure 8:**
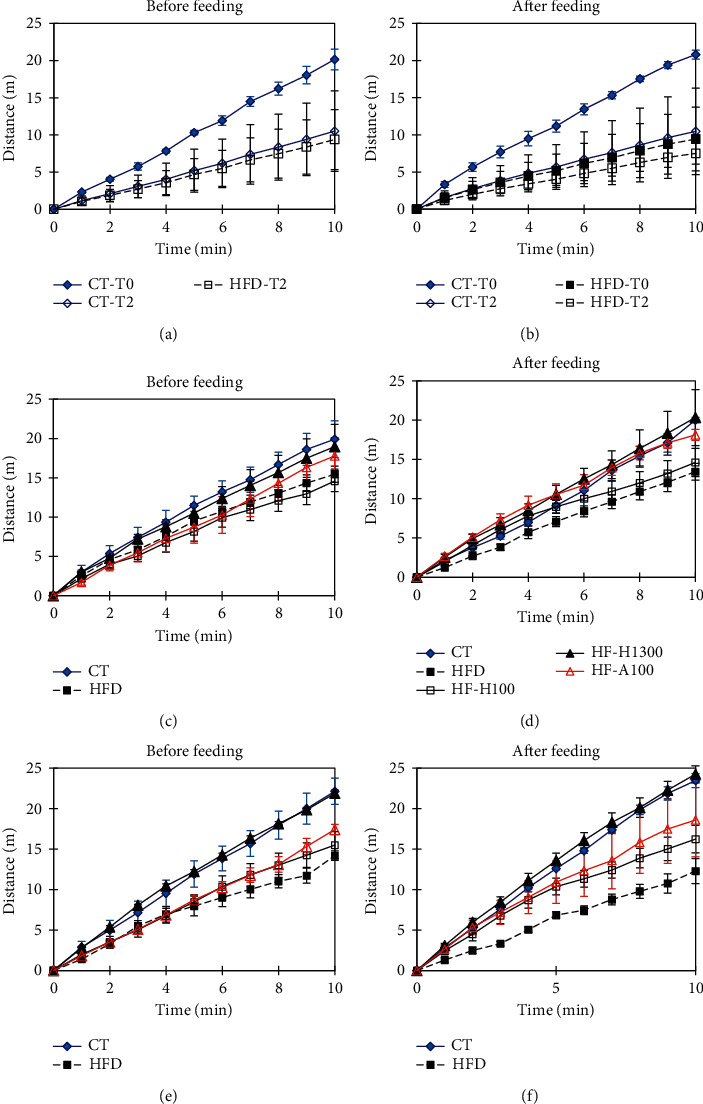
The travel distance of tested mice before (a) and after (b) feeding, (A and B T0 and T2 are week 0 and 2, respectively; C and D are week 4; E and F are week 6) (data were shown in mean ± standard deviation, *n* = 5).

Figure 8 shows the travel distance (TD) of the mouse groups before and after feeding at weeks 0, 2, 4, and 6. The HFD group always had a much lower TD than one of the CT group both before and after feeding at all levels. Before and after eating, the CT group had fairly similar TD values (except for week 6, where before-feeding-TD was slightly higher than after-feeding-TD). In contrast, in the HFD group, after-feeding-TD was consistently lower than before-feeding-TD.

The HF–H100 and HF–H1300 groups (respectively, the lowest and highest doses of Duong-dau root extract in our study) were selected to evaluate the locomotion activity. The results showed that the TDs of the groups were ranked in the following order: CT≈HF-H1300 > HF-A100 > HF-H100 > HFD (both before and after feeding). Thereby, the results showed that the higher the dose was, the better the mobility was. Apparently, over the test period, the obese mice (HFD) moved less and less. This is also observed in previous studies [63, 64]. According to Gupta [65], type 2 diabetes is often associated with anxiety. In addition, insulin (a peptide hormone) has an important function in the brain and helps alleviate some of the psychological problems caused by diabetes. Therefore, the use of higher doses of extracts will help prevent type 2 diabetes, increase insulin regulation, reduce anxiety and improve the performance of mice.

**Figure 9:**
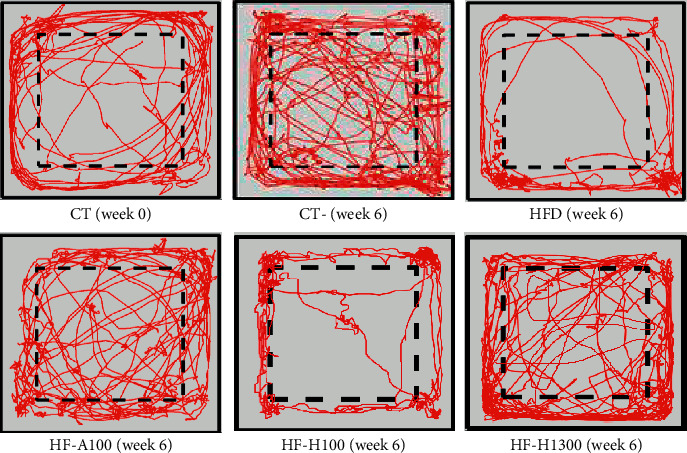
Representative trajectories of test mice. Note: CT-T0 and CT-T6 are CT groups at week 0 and week 6. The peripheral region is the space between solid and dashed lines. The central area is the space inside the dashed line.

Figure 9 shows the representative trajectory of the groups of mice after 6 weeks of testing. In the case of the CT group at the start time (week 0) and the end time (week 6), their moving areas were not different, they traveled all positions in the open field (OF). At week 6, compared with the other groups, the HFD group had less travel distance and most of the migration was only in the peripheral region of the OF. The migration route of the HF-H100 and HF-A100 groups went into the central area more than that of the HFD group. At the dose of HF-H1300, the immigration and travel distance were quite similar to the CT group, they moved evenly in the survey area in both the periphery and the center. Our results showed that mice that were overweight and obese as well as have poor glycemic and blood lipid control tend to be less mobile, with migration routes predominantly in the peripheral region (caused by the thigmotaxis which is anxious or scared) [66]. Meanwhile, groups of mice that received AIG-supplemented diets had higher TDs and immigration was also diverse with a higher degree of radial. In summary, the use of Duong-dau root extract and acarbose improved the movement and psychological well-being of mice. Accordingly, the higher the dose was, the greater the possibility of improvement.

In addition, this study also evaluated the ability of the extract to kill experimental animals at extremely high doses. After 3 months of administration at a dose of 5000 mg/kg body weight/day (equivalent to 350 g/day for a 70 kg human), no animals appeared abnormal or died. This initially demonstrated the safety of the extract.

## 4. Conclusion

In this study, the use of Duong-dau extract in a high-fat diet helped (a) significantly reduce the glycemic index, and body weight of tested mice; (b) reduce TG, TC/HDL, LDL/HDL index, increase HDL in the blood, limit the risk of cardiovascular disease; (c) histopathological studies of white adipose, liver, kidney tissues as well as locomotion activity have confirmed the abovementioned advantages of this extract. Thus, *n*-hexane extract from the roots of Duong-dau tree has proven to be strongly biologically active in preventing and supporting the treatment of diseases related to overweight and obesity, helping to control blood glucose levels thereby reducing the risk of type 2 diabetes. In the future, we propose to separate the extract into different fractions to further evaluate *in vivo*. Simultaneously, step-by-step preparation of this extract to commercial products in the form of functional foods or drugs derived from medicinal herbs.

## Figures and Tables

**Table 1:** Diets of tested mice.

Group	Feeding
CT^1^	Control diet (composition (g/100 g): total carbohydrate of 70.8; protein of 15.5; lipid of 3.26; crude fiber of 16.0; total energy (kcal) of 374.54)
HFD^2^	High-fat diet (composition (g/100 g): total carbohydrate of 49.56; protein of 10.85; lipid (include of beef tallow) of 32.28; crude fiber of 11.2; total energy (kcal) of 532.18)
HF–H100	HFD + 2 × 50 mg extract/kg of body weight/day
HF–H500	HFD + 2 × 250 mg extract/kg of body weight/day
HF–H900	HFD + 2 × 450 mg extract/kg of body weight/day
HF–H1300	HFD + 2 × 650 mg extract/kg of body weight/day
HF-A100	HFD + 2 × 50 mg acarbose/kg of body weight/day

^1^A commercial product (FULLVITA JP04, based on AIN-93 diet) [19]; ^2^beef tallow (30 g) was boiled and mixed with CT (70 g). Then, HFD was kept in a zip-lock plastic bag at 4°C for further experiment.

**Table 2:** Blood lipid.

	Group	TG (mmol/l)	TC (mmol/l)	HDL (mmol/l)	LDL (mmol/l)	TG/hdl^*∗*^	TC/hdl^*∗*^	LDL/hdl^*∗*^
Week 0	CT	1.05 ± 0.10^c^	2.36 ± 0.21^a^	1.39 ± 0.02^a^	0.8 ± 0.01^a^	0.76^f^	1.70^c^	0.58^b^
	CT	1.15 ± 0.02^d^	2.83 ± 0.19^b^	1.66 ± 0.10^c^	0.9 ± 0.00^a^	0.69^e^	1.70^c^	0.54^b^
	HFD	1.77 ± 0.09^g^	3.97 ± 0.25^e^	1.52 ± 0.09^b^	1.8 ± 0.09^d^	1.16^g^	2.61^e^	1.18^d^
	HF-H100	1.23 ± 0.11^e^	3.84 ± 0.29^d^	2.06 ± 0.12^d^	1.4 ± 0.07^c^	0.60^d^	1.86^d^	0.68^c^

Week 6	HF-H500	0.92 ± 0.01^b^	3.59 ± 0.18^c^	2.13 ± 0.21^e^	1.2 ± 0.12^b^	0.43^c^	1.69^bc^	0.56^b^
	HF-H900	0.8 ± 0.01^a^	3.6 ± 0.31^c^	2.17 ± 0.08^f^	1.1 ± 0.04^b^	0.37^b^	1.66^b^	0.51^b^
	HF-H1300	0.76 ± 0.05^a^	3.52 ± 0.02^c^	2.37 ± 0.18^g^	0.9 ± 0.03^a^	0.32^a^	1.49^a^	0.38^a^
	HF-A100	1.59 ± 0.01^f^	3.63 ± 0.11^c^	2.12 ± 0.05^e^	1.2 ± 0.06^b^	0.75^f^	1.71^c^	0.57^b^

TC: total cholesterol; TG: triglycerides; HDL: high-density lipoprotein cholesterol; and LDL: low-density lipoprotein cholesterol. The results are expressed as mean ± standard deviation (*n* = 5). Numbers in a column with different superscript letters are significantly different (*p* < 0.05). ^*∗*^The ratio of means.

**Table 3:** Weight of tissues after 6 weeks of the experiment.

Group	LW (g)	LW/BW (%)	KW (g)	KW/BW (%)	AW (g)	AW/BW (%)
CT	1.28 ± 0.02^a^	3.45 ± 0.07^a^	0.32 ± 0.01^a^	0.85 ± 0.01^a^	0.66 ± 0.10^a^	1.78 ± 0.30^a^
HFD	1.58 ± 0.04^b^	3.68 ± 0.03^b^	0.43 ± 0.02^b^	1.00 ± 0.04^b^	2.00 ± 0.13^d^	4.65 ± 0.26^d^
HF-H100	1.42 ± 0.06^a^	3.53 ± 0.06^a^	0.35 ± 0.01^a^	0.87 ± 0.02^a^	1.49 ± 0.15^c^	3.72 ± 0.26^c^
HF-H500	1.36 ± 0.03^a^	3.53 ± 0.02^a^	0.35 ± 0.02^a^	0.89 ± 0.03^a^	1.36 ± 0.09^c^	3.52 ± 0.19^c^
HF-H900	1.31 ± 0.03^a^	3.52 ± 0.07^a^	0.34 ± 0.02^a^	0.90 ± 0.04^a^	1.20 ± 0.08^cd^	3.23 ± 0.13^bc^
HF-H1300	1.29 ± 0.05^a^	3.49 ± 0.05^a^	0.32 ± 0.01^a^	0.87 ± 0.03^a^	0.94 ± 0.06^ab^	2.54 ± 0.10^ab^
HF-A100	1.37 ± 0.05^a^	3.52 ± 0.02^a^	0.37 ± 0.02^a^	0.95 ± 0.04^ab^	1.49 ± 0.18^c^	3.83 ± 0.46^cd^

LW: liver weight; BW: body weight; KW: kidney weight; and AW: adipose tissue weight. The results are expressed as mean ± standard deviation (*n* = 5). Numbers in a column with different superscript letters are significantly different (*p* < 0.05).

## Data Availability

The data supporting the results of this study are included in the manuscript.
